# Validation of canine uterine and testicular arteries for the functional characterisation of receptor-mediated contraction as a replacement for laboratory animal tissues in teaching

**DOI:** 10.1371/journal.pone.0230516

**Published:** 2020-05-26

**Authors:** Louise Mulcahy, Elizabeth Tudor, Simon R. Bailey

**Affiliations:** Department of Veterinary Biosciences, Melbourne Veterinary School, Faculty of Veterinary and Agricultural Sciences, University of Melbourne, Parkville, Victoria, Australia; University of Southampton, UNITED KINGDOM

## Abstract

Teaching practicals for receptor physiology/pharmacology in medical and veterinary schools have involved the use of *in vitro* experiments using tissues from laboratory animals, which have been killed for isolated vascular strip or ring preparations. However, the use of scavenged tissues has been advocated to reduce animal use. Utilising discarded tissues from routine surgical procedures, such as canine neutering, has not previously been investigated. Canine testicular and uterine tissues (discarded tissues) were obtained from routine neutering procedures performed by the veterinary team at a local animal neutering clinic for stray dogs. Rings of uterine and testicular artery were dissected and mounted on a Mulvany-Halpern wire myograph in order to characterize the adrenergic and serotonergic receptors mediating vasoconstriction. Cumulative contractile concentration-response curves were constructed for the alpha adrenoceptor agonists epinephrine (α_1_ and α_2_ receptors), phenylephrine (α_1_ selective) and UK14304 (α_2_ selective). Pre-treatment with the α_1_-selective antagonist, prazosin, was also investigated. The response to serotonin (5-HT) receptor agonists were also investigated, including 5-HT (acting at both 5-HT_1_ and 5-HT_2_ receptors), 5-carboxamidotryptamine (5-CT; 5-HT_1_ selective) and α-methyl 5-HT (5-HT_2_ selective). A contractile response was observed in both canine uterine and testicular arteries to epinephrine and phenylephrine, and prazosin caused a dose-dependent parallel rightward shift in the phenylephrine dose-response curve (pA_2_ values of 7.97 and 8.39, respectively). UK14304 caused a contractile response in canine testicular arteries but very little appreciable contractile response in uterine arteries. The maximum responses produced by the uterine arteries to 5-HT was significantly lower than those of the testicular arteries. In the testicular artery, the 5-HT_2_ receptor selective agonist, α-methyl 5-HT, produced a similar contractile response to 5-HT but the administration of 5-CT failed to produce a response in either the testicular or uterine artery segments. These results validate the use of discarded tissue from routine canine neutering procedures as a useful source of vascular tissue for pharmacological teaching, for characterizing alpha and 5-HT receptor contractile responses.

## Introduction

Domestic animal species such as dogs are still widely used for drug design and development, toxicity testing, research and education [1]. Their use has provided a great deal of knowledge about animal physiology and disease, the pharmaco-kinetics and pharmacodynamics of pharmaceutical compounds, and the development and application of many procedures and devices [2]. This information has been extrapolated to provide information relevant to many fields of science, including human medicine, veterinary medicine, pharmacy and dentistry [2]. With an evolving societal awareness of animal welfare, the use of these and other animal species in research has been questioned and debated over many decades. This has culminated in the development and implementation of the principle ‘reduce, refine and replace’ in relation to scientific animal use [3].

The principles of ‘the 3Rs’ (Replacement, Reduction and Refinement) were developed over 50 years ago providing a framework for performing more humane animal research [3]. Since then they have been embedded in national and international legislation and regulations on the use of animals in scientific procedures, as well as in the policies of organisations that fund or conduct animal research. For certain areas of pharmacology, all three principles cannot be applied, e.g. the principle of replacement is difficult to achieve with regulatory toxicology [4]. In contrast, the area of teaching and education is one where all three principles can, and should, be applied.

Traditionally, teaching practicals for receptor physiology/pharmacology in medical and veterinary schools have involved the use of *in vitro* experiments using tissues from laboratory animals, which have been killed for isolated vascular strip or ring preparations [1]. New methods have been developed in an attempt to reduce animal tissue use. These include scavenged tissue (tissues from deceased animals), secondary use (secondary procedure performed on an animal under anaesthesia) and re-use (individuals/cohorts of animals used as both control and treatment groups) [5]. One method that is not widely described in the literature is utilising discarded tissues from routine surgical procedures, such as neutering. This method of tissue collection does not compromise the animal and is a novel embodiment of the ‘reduce’ principle. This paper utilises this novel source of animal tissue.

Strip preparations of vascular tissue have long been used to demonstrate drug-receptor interactions [6]. In a teaching setting, organ baths are commonly used due to convenience as they facilitate the use of larger vessels [7], while in contrast wire myographs are commonly used in research [7]. It was Mulvany & Halpern [8] who first described a method for examining vessels of 100–500 mm in diameter, which more closely represent resistance vessels rather than conducting vessels. Their method has since been refined, and customised equipment developed, to study the function of these vessels. In this study, the technique of wire myography has been used to classify the post-junctional receptors (adrenoceptors and 5-HT receptors) mediating vasoconstriction in canine testicular and uterine arteries. These muscular arteries are physiologically quite distinct from elastic arteries such as the rat aorta that is commonly used to teach vascular physiology and pharmacology.

The literature has described both adrenoceptors and 5-HT receptors causing a vasoconstrictive response in canine arteries [9, 10]. Characterising these particular blood vessels will facilitate the use of this tissue (usually discarded following routine neutering procedures of pet animals) for the teaching of receptor physiology and pharmacology in veterinary and non-veterinary courses. It is also considered that this may serve as a validated source of tissue in which to replace killed animals in a university setting.

## Materials and methods

### Vascular tissues

This study involved no live animals, only the secondary use of discarded animal tissues. The Animal Ethics Committee of the University of Melbourne approved the use of scavenged tissues for this study (formal waiver of ethical approval).

Canine testicular and uterine tissues were obtained from routine neutering procedures performed by the veterinary team at a local animal neutering clinic for stray dogs. The tissues were collected from healthy dogs of various ages and breeds. The dogs were not being used for research purposes, but were intended to be adopted after recovery from surgery.

Tissues were placed in ice-cold physiological Krebs solution for transportation.

Second order branches of the uterine artery and the distal region of the testicular artery, representing resistance arteries, were dissected from surrounding tissue using a dissecting microscope. Segments (1-2mm) were placed in physiological Krebs solution and stored at 4°C for up to 48 hours.

### Wire myography

The segments were mounted into a Mulvany-Halpern wire myograph using wire of 40 μm diameter (Danish Myo Technologies, Denmark). Physiological Krebs-Henseleit solution (5mL) bathed the tissues in the myograph. The Krebs solution contained 118.0mM/L NaCl, 4.7mM/L KCl, 1.2 mM/L MgSO_4_, 1.2mM/L KH_2_PO_4_, 25.0 mM/L NaHCO_3_, 11.1mM/L D-glucose and 2.5mM/L CaCl_2_.

Arteries were maintained in oxygenated (95%O_2_ and 5%CO_2_) Krebs solution at 37°C. Preparations were allowed to equilibrate for 30 minutes before a normalization procedure was performed to determine the optimal internal circumference and to normalize the resting tension [7]. A passive length-tension relationship was described by determining the vessel radius at which the wall tension was equivalent to 100mmHg and the tension was subsequently set to achieve this value. After a further period of equilibration, the maximum contractile response to a depolarizing stimulus was obtained by replacing the Krebs solution with an equivalent solution containing 118 mM KCl (Depolarising Kreb’s solution; DKS). Artery segment contraction was expressed as mN per mm segment length and continuously recorded by a computerised acquisition system (Power Lab, ADI Instruments, Oxfordshire, UK).

### Receptor agonists and antagonists

The drugs used in the experiment included alpha adrenoceptor agonists epinephrine (acting at both α_1_ and α_2_ receptors), phenylephrine (α_1_ selective) and UK14304 (α_2_ selective). Pre-treatment with the α_1_-selective antagonist, prazosin (10^-8^M or 10^-7^M), was also used. Serotonin (5-HT) receptor agonists were also used in the study, including 5-HT (acting at both 5-HT_1_ and 5-HT_2_ receptors), 5-carboxamidotryptamine (5-CT; 5-HT_1_ selective) and α-methyl 5-HT (5-HT_2_ selective). Stock solutions of each drug were produced by dissolving the drug in distilled water. The concentrations presented in the results represent the final molar concentration in the bathing medium. All drugs were obtained from Sigma-Aldrich Ltd (Sydney, NSW).

### Concentration-response curves and data analysis

After a period of stabilization of resting tension, agonists were added in a cumulative manner. The tissues were allowed to reach maximum contraction for that agonist concentration, i.e. contract until a steady state was obtained, before the next concentration was added. For the antagonist experiments, the preparations were incubated with the antagonist for 10 mins before agonist concentrations were added to the bathing medium. Blood vessel segments from 4–6 individual animals were used for each treatment, and the effects of antagonists were examined on paired segments from the same animal.

The contractile response to each concentration of agonist was determined for each tissue and expressed as a percentage of the contractile response produced by the depolarizing stimulus (% DKS). A curve-fitting program (Graph Pad Prism, Version 6.02) was used to calculate the maximal response value for each individual curve. The equation used to fit the monophasic concentration-response curves was: E = [E_max_ A^nH^/ (A^nH^ + EC_50_)], where E_max_ is the maximum response and nH represents the Hill slope.

Statistical analysis was performed on the data using Graphpad Prism (Version 6.02; Graphpad Inc.). A Kruskal-Wallis test was used to compare the values obtained from the testicular artery 5-HT, alpha-methyl 5-HT and 5-CT treatments and a Mann-Whitney test was used on all other data. Statistical significance was accepted at a P-value of <0.05.

## Results

### Tissue characteristics and force generated

The internal diameter of the uterine and testicular artery segments ranged from 357–1,570 micrometers and 190–716 micrometers, respectively. The maximum tension generated by the uterine artery segments to depolarising Krebs solution reached 2–12 grams, while the testicular artery segments generated 2–8.8 grams tension.

### Characterisation of alpha adrenoceptors

**Fig 1:**
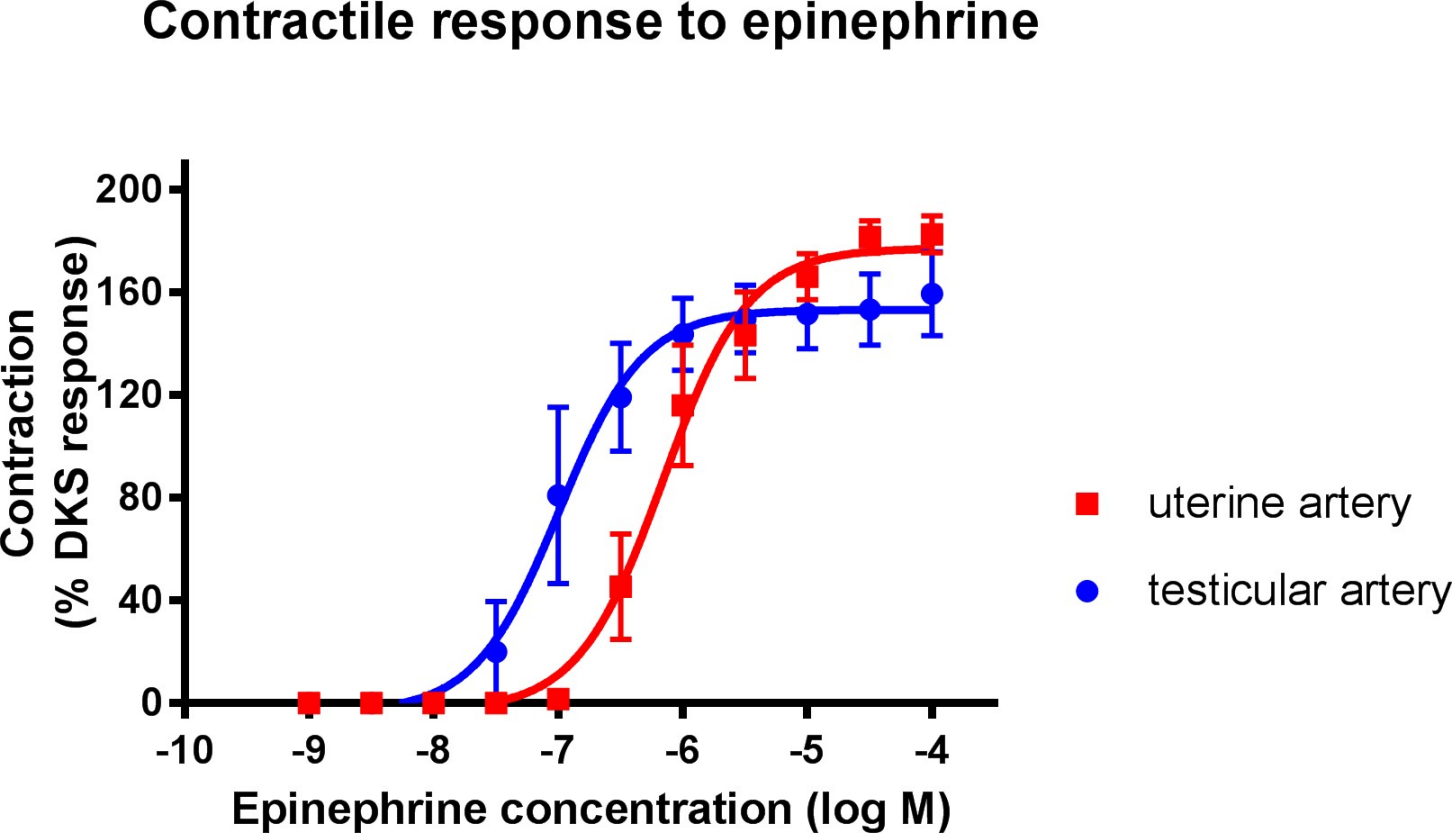
Concentration-response curves for epinephrine in isolated canine uterine arteries (red squares) and testicular (blue circles) arteries. Each point represents the mean ± SEM.

A contractile response was observed in both canine testicular and uterine arteries with the administration of the non-selective alpha adrenoceptor agonist, epinephrine (Fig 1). The EC_50_ values, maximum responses and Hill slope values for all of the experiments are provided in Tables 1 and 2. A statistically significant difference was noted in the EC_50_ values between uterine (1.1 ± 0.55 x10^-6^M) and testicular arteries (1.51 ± 0.50 x10^-7^M); however, there was no significant difference in the maximum response in either tissue, when accounting for the vessel size by expressing the tension as a percentage of the DKS response.

**Table 1:** Concentration-response curve parameters derived by curve fitting from the contractile responses to adrenoceptor and 5-HT receptor agonists obtained from canine uterine arteries.

Treatment	EC_50_ (M)	Max response (%DKS)	Hill slope
	(mean ± sem)	(mean ± sem)	(mean ± sem)
Uterine artery epinephrine	1.10 ± 0.55 x10^-6^ M	178.38 ± 9.75	1.84 ± 0.33
Uterine artery PE	2.41 ± 1.47 x10^-6^ M^a^	167.20 ± 35.35	1.30 ± 0.30
Uterine artery PE+ prazosin (100nM)	1.66 ± 0.06 x10^-5^ M^a^	145.80 ± 6.41	1.18 ± 0.16
Uterine artery UK14304	ND	4.18 ± 0.51	ND
Uterine artery 5-HT	2.26 ± 1.93 x10^-6^ M	51.88 ± 17.67	2.88 ± 0.80

Values with the same superscript letter are significantly different from each other, as described below. There was minimal response to the α_2_ adrenoceptor agonist UK14304 in uterine arteries and therefore EC_50_ values were not determined (ND).

^a^ = significant difference uterine artery phenylephrine vs. phenylephrine + prazosin (100 nM), P = 0.016

**Table 2:** Concentration-response curve parameters derived by curve fitting from the contractile responses to adrenoceptor and 5-HT receptor agonists obtained from canine testicular arteries.

Treatment	EC_50_ (M)	Max response (%DKS)	Hill slope
	(mean ± sem)	(mean ± sem)	(mean ± sem)
Testicular artery epinephrine	1.51 ± 0.50 x10^-7^ M^a^	150.65± 11.91	2.00 ± 0.16
Testicular artery PE	1.30 ± 0.75 x10^-6^ M^b^^,^^c^	123.97 ± 20.08	2.74 ± 0.64
Testicular artery PE + prazosin	3.31 ± 1.30 x10^-5^ M^b^	188.30 ± 23.73	2.23 ± 0.20
Testicular artery UK14304	7.63 ± 3.24 x10^-8^ M^c^^,^^d^	123.48 ± 28.99	1.95 ± 0.49
Testicular artery 5-HT	2.61 ± 1.91 x10^-6^ M	118.86 ± 16.51^e^^,^^f^	2.21 ± 0.21
Testicular artery α-methyl 5-HT	1.01 ± 0.55 x10^-6^ M	92.73 ± 16.11^f^	1.29 ± 0.06
Testicular artery 5-CT	ND	0 ± 0^f^	ND

Values with the same superscript letter are significantly different from each other, as described below. Differences between the responses of the testicular arteries and uterine arteries to the same agonists are also indicated. There was minimal response to the 5-HT_1_ receptor agonist 5-carboxamidotryptamine (5-CT) in testicular arteries and therefore EC_50_ values were not determined (ND).

^a^ = significant difference uterine artery epinephrine vs. testicular artery epinephrine, P = 0.029

^b^ = significant difference testicular artery phenylephrine vs. phenylephrine + prazosin (100 nM), P = 0.016

^c^ = significant difference testicular artery phenylephrine vs. UK14304, P = 0.032

^d^ = significant difference uterine artery vs. testicular artery UK14304, P = 0.001

^e^ = significant difference uterine artery 5-HT vs. testicular artery 5-HT, P = 0.032

^f^ = significant difference testicular artery 5-HT vs. α-methyl 5-HT vs. 5-CT, P = 0.045

**Fig 2:**
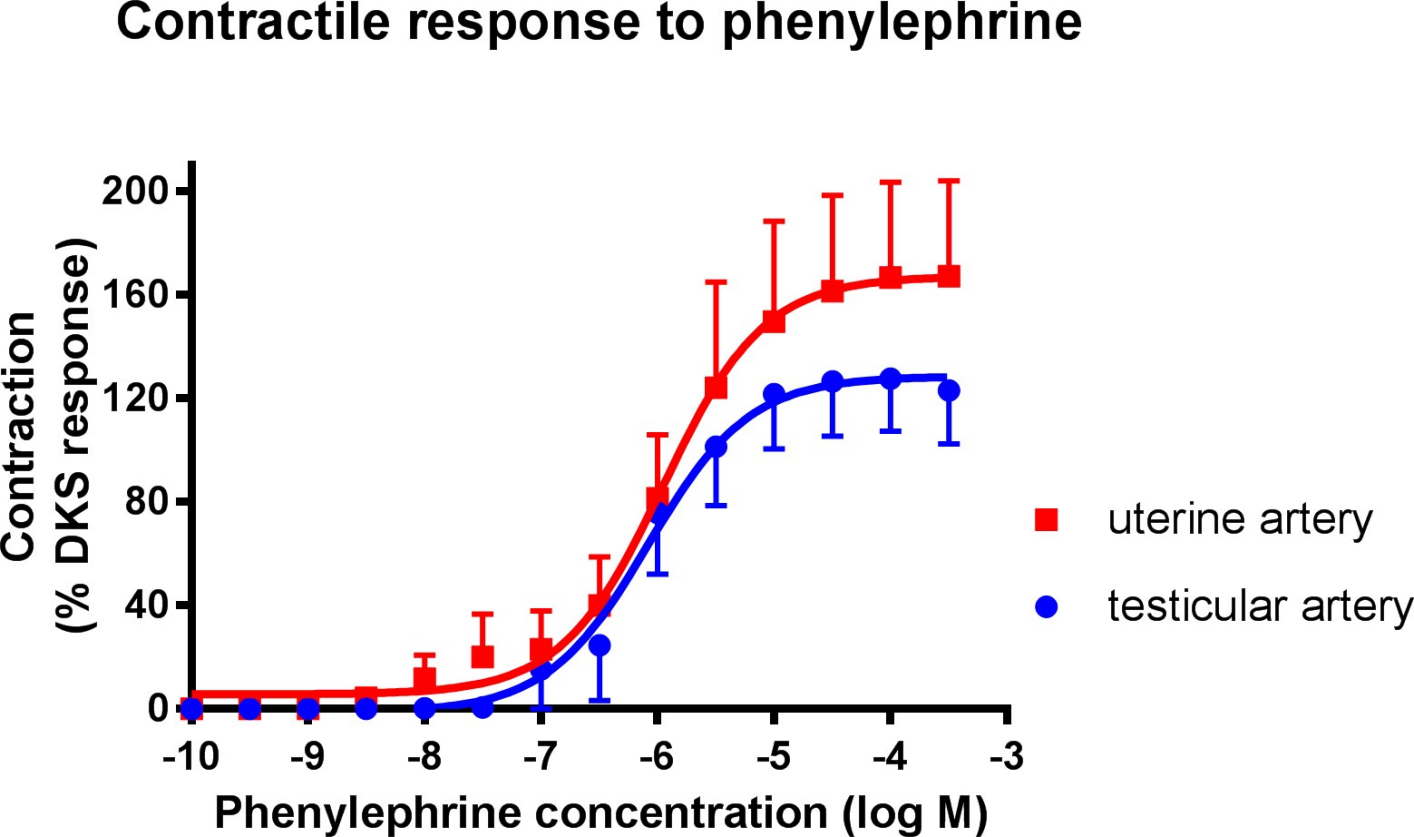
Concentration-response curves for phenylephrine (PE) in isolated canine uterine arteries (red squares) and testicular (blue circles) arteries. Each point represents the mean ± SEM.

**Fig 3:**
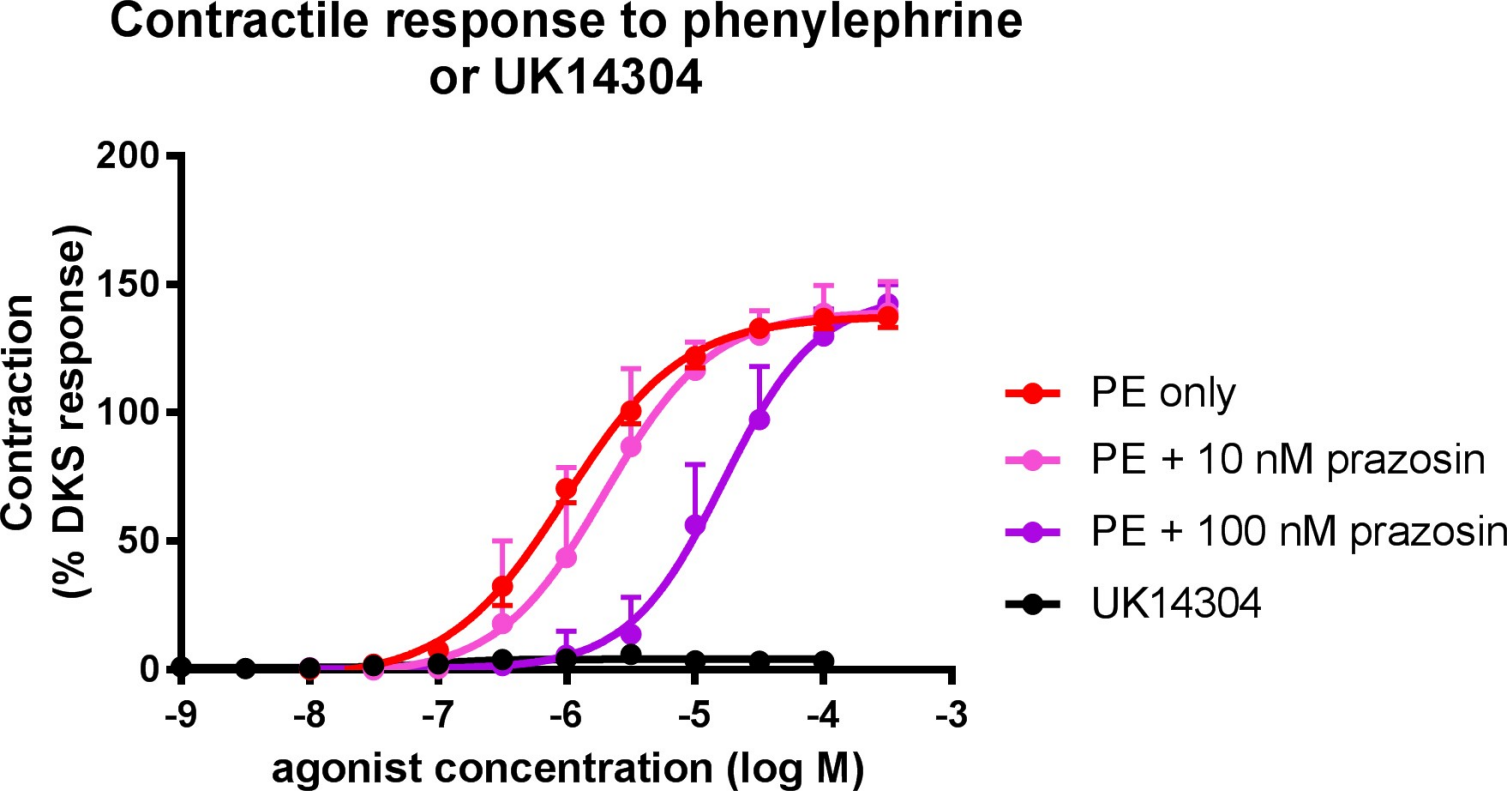
Concentration-response curves for the alpha-2 adrenoceptor agonist, UK14304 (black), and phenylephrine (PE) in the absence (red) and presence of prazosin (10 nM or 100 nM; pink and purple, respectively) in isolated canine uterine arteries. Each point represents the mean ± SEM.

**Fig 4:**
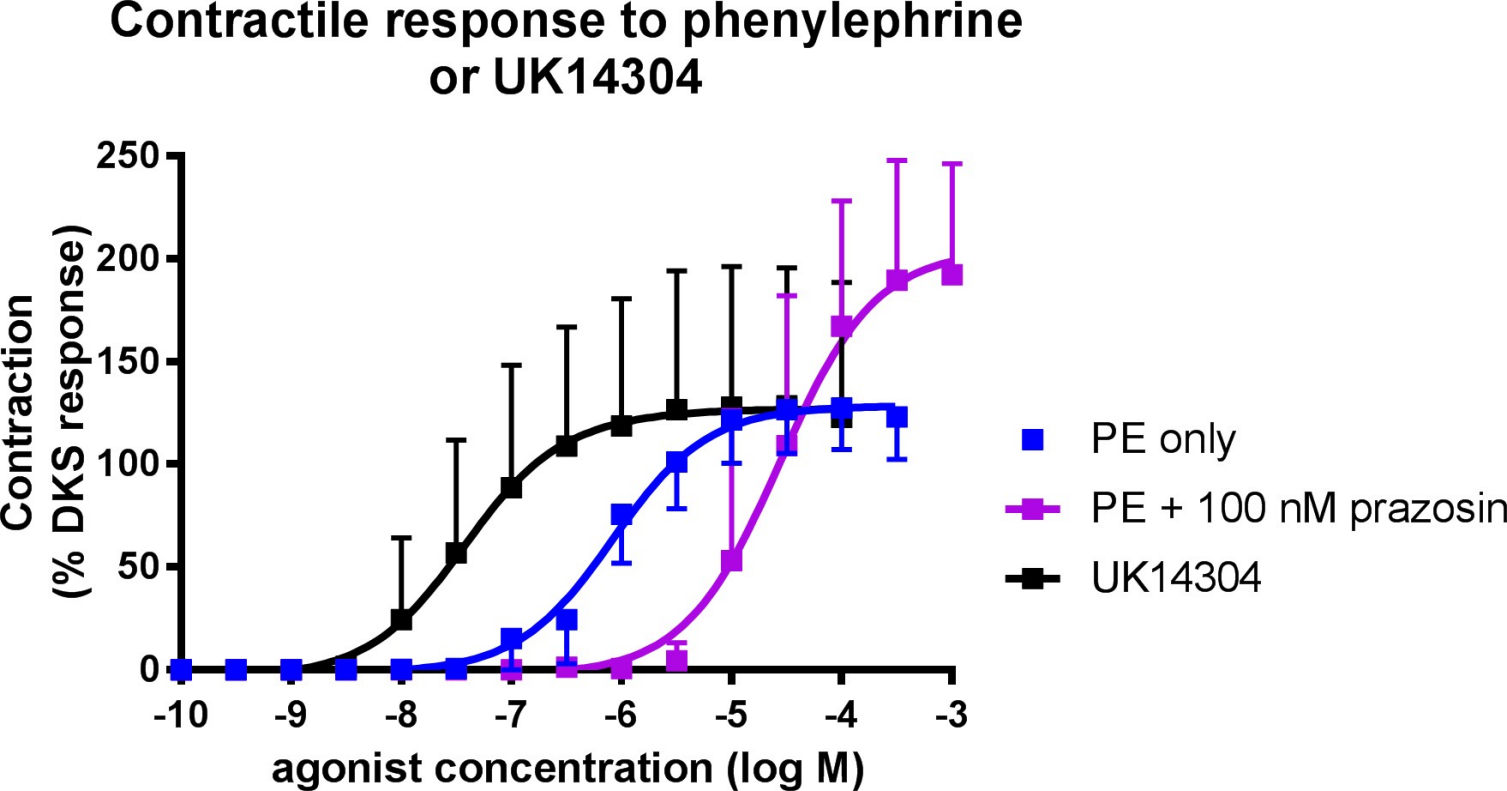
Concentration-response curves for the alpha-2 adrenoceptor agonist, UK14304 (black), and phenylephrine (PE) in the absence (blue) and presence of prazosin (100 nM; purple) in isolated canine testicular arteries. Each point represents the mean ± SEM.

Phenylephrine administration (alpha_1_ selective agonist) produced a contractile response in both uterine and testicular arteries (Fig 2). No significant difference was observed between the tissues in regards to EC_50_ or maximum response (Tables 1 and 2). Pre-treatment of canine uterine arteries with the alpha_1_ selective antagonist, prazosin (10 and 100 nM), caused a dose-dependent parallel rightward shift in the phenylephrine dose-response curve (Fig 3). This produced a pA_2_ value of 7.97 for prazosin, which is consistent with its actions on a population of α_1_ adrenoreceptors. Pre-treatment of canine testicular arteries with prazosin (100 nM), also caused a significant rightward shift (25-fold) in the phenylephrine dose-response curve (Fig 4), giving an apparent pA_2_ value of 8.39. In both the uterine and testicular arteries, there was no significant effect of prazosin on the maximum responses produced by phenylephrine.

There was very little appreciable contractile response to the selective alpha_2_ adrenoceptor agonist, UK14304, in canine uterine arteries (Fig 3). A maximum response of 4.18 ±0.51% DKS was recorded, but this response was too small to be curve-fitted. However, a dose-dependent strong contractile response was observed in canine testicular arteries to UK14304 (Fig 4), with a maximum response very similar to phenylephrine. A statistically significant difference was seen in the EC_50_ values between phenylephrine (1.3 ± 0.75 x10^-6^M) and UK14304 (7.63 ± 3.24 x10^-8^M) administration.

### 5-HT receptor characterisation

**Fig 5:**
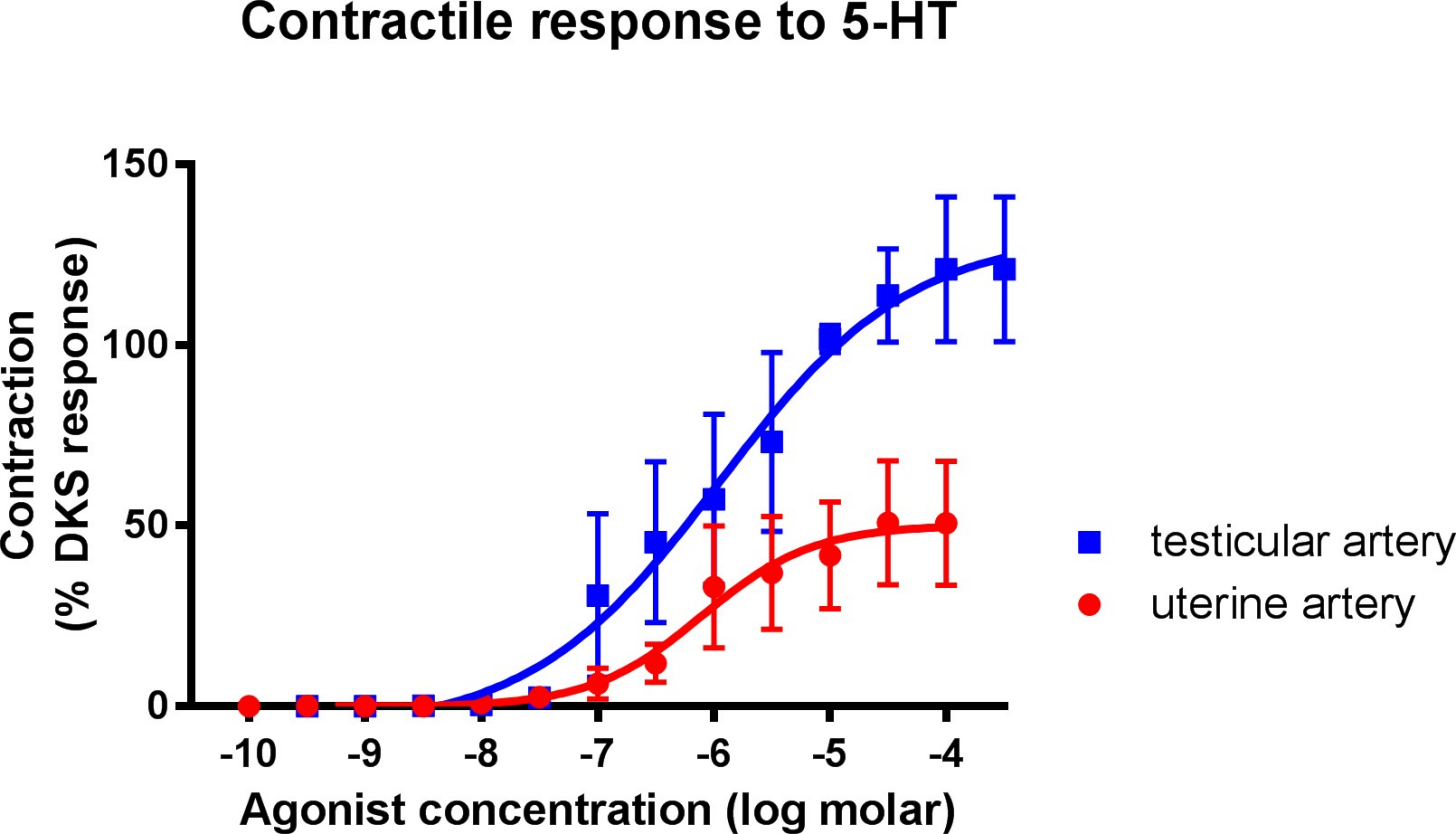
Concentration-response curves for 5-HT in isolated canine uterine (red circles) and testicular (blue squares) arteries. Each point represents the mean ± SEM.

Both uterine and testicular arteries produced a contractile response to 5-HT administration (Fig 5). There was no significant difference in the EC_50_ value for either tissue. However, the maximum responses produced by the uterine arteries was significantly lower than those of the testicular arteries when expressed as a percentage of the DKS response (mean of 51.88 ± 17.67% of the DKS response for the uterine arteries and 118.86 ± 16.51% DKS for the testicular arteries).

**Fig 6:**
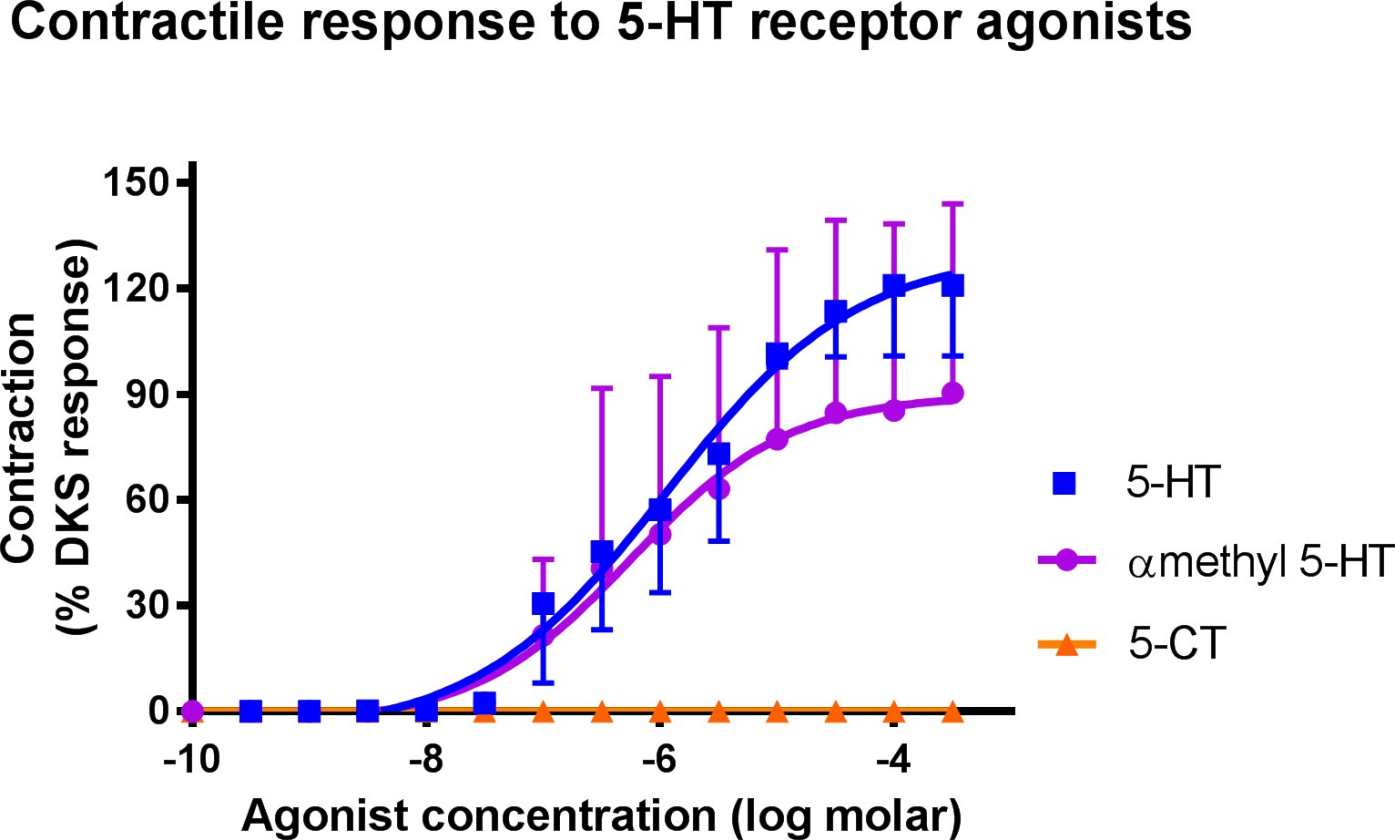
Concentration-response curves for 5-HT (blue squares), α-methyl 5-HT (purple circles) and 5-carboxamidotryptamine (5-CT; orange triangles) in isolated canine testicular tissue. Each point represents the mean ± SEM.

In the testicular artery, the 5-HT_2_ receptor selective agonist, α-methyl 5-HT, produced a similar contractile response to 5-HT (Fig 6). In contrast, the administration of 5-CT failed to produce a response. A statistically significant difference was seen in the maximum responses produced by 5-HT, α-methyl 5-HT and 5-CT (118.86 +/- 16.51% DKS, 92.73 +/- 16.11% DKS and 0 +/- 0% DKS respectively). In the uterine artery segments, 5-CT also failed to produce a response.

In the testicular artery, it was possible to fit the data from the 5-HT experiment to a biphasic model, giving an EC_50_ value for the first phase of 7.1 and the second phase 5.3. However, the R squared value for the goodness of fit for the biphasic model (0.74) was not significantly different to that obtained from the monophasic model (0.73). Therefore it could not be definitively determined whether two different receptor subtypes may have been mediating this response.

## Discussion

This study investigated the post-junctional α-adrenergic and 5-HT receptors mediating vasoconstriction in canine uterine and testicular arteries, in order to characterise the receptor populations to see how these blood vessels might be useful for teaching purposes. This study demonstrated that α-adrenergic and 5-HT receptors are present in both canine and testicular arteries, with further characterisation conducted to determine the subtypes. It also demonstrated that the use of discarded tissues from routine neutering procedures is a valid and useful source of experimental tissue. The amount of force generated by the muscular canine uterine and testicular arteries makes them superior tissues for teaching, with force generation of up to 12 and 8.8 grams tension, respectively. Rat aorta is a commonly used tissue for teaching, but this is an elastic artery and equivalent sized rings can only generate around 1.5–2 grams tension [11, 12].

In both tissue types, a vasoconstrictive response was observed in a dose-dependent manner to epinephrine, a non-selective adrenoceptor agonist [13], demonstrating the adrenoceptor-mediated contractile response to the natural ligand. As there was no significant difference between the maximum response produced by both tissues, it can be assumed that epinephrine is equally efficacious in both canine uterine and testicular arteries. The variation in EC_50_ values suggests that epinephrine may be more potent in the canine testicular artery compared to the uterine artery. It has been shown that different vascular beds have marked differences in their responsiveness to epinephrine, and other vasoactive substances, likely the result of different receptor populations or receptor sensitivity [14]. One potential explanation is the presence of oestrogen on uterine arteries. Oestrogen has been shown to alter ion fluxes in arterial walls and also modulate endothelium-derived factors [15]. An inhibitory effect on smooth muscle is observed *in vivo* by oestrogen via inhibiting calcium influx and activating potassium efflux [15]. Other studies have also commented on sexually dimorphic vascular responses [16, 17].

To further characterise the adrenoceptor population present in both the uterine and testicular tissue an α_1_-selective agonist [13], phenylephrine, was administered. A vasoconstrictive response in both tissues occurred in a dose-dependent manner. Previous studies have demonstrated that canine arterial smooth muscle contains α1-adrenoceptors [18]. As no significant difference was seen in the response to phenylephrine on these tissues, it may be presumed that the α_1_ receptor population is similar in both uterine and testicular arteries. This is an interesting finding as previous studies have shown a variation in α_1_-adrenoceptor expression as a result of oestrogen presence [19]. Studies have also shown a significant decrease in the maximal arterial vasoconstrictive response produced by phenylephrine in rats treated with 17β-estradiol [17, 20].

In both the uterine and testicular tissues, incubation with prazosin, an α_1_-selective antagonist [13], during the administration of phenylephrine demonstrated competitive inhibition by causing a parallel rightward shift in the concentration-response curves. This competitive inhibition, with pA_2_ values of 7.97 and 8.39, further confirms the presence of α_1_ receptors mediating vasoconstriction in canine uterine and testicular arteries, because these values are consistent with the affinity of prazosin at α_1_-adrenoceptors in previously characterised tissues [21].

The administration of UK14304, an α_2_-selective agonist [22], produced almost no contractile response in the canine uterine artery segments, suggesting that very few (if any) α_2_ adrenoceptors are present in these blood vessels. However in testicular arteries this agonist produced a very clear dose-dependent vasoconstrictive response. In fact, both phenylephrine and UK14304 were similarly efficacious in these arteries. This demonstrates the presence of a mixed population of α-adrenoceptors in the testicular arteries (both α_1_ and α_2_ adrenoceptors). The high efficacy and potency of UK14304 suggests that there is a significant population of α_2_-adrenoceptors in these arteries, although in order to determine the relative functional significance of this receptor population the effect of an α_2_-adrenoceptor-selective antagonist would need to be examined on the contractile response to a non-selective α agonist (e.g. epinephrine). With an EC_50_ value of 76 nM, it can be presumed that UK14304 is acting at the α_2_ receptor population and not activating α_1_ receptors non-selectively, which can occur with selective agonists administered at high concentrations [13].

There are limited studies evaluating receptor classification in testicular arteries, particularly in the canine model. However, one study indicated that α_2_-adrenoceptors likely contribute to smooth muscle contraction in the rat testicular capsule [23]. As it has been shown that canine vessels in different body regions have varied α-adrenoceptor subtypes [24], it is likely that α_2_-adrenoceptors may be more prominent in canine testicular arteries. Relative proportions of alpha adrenoceptors (α_1_ and α_2_) vary depending on the diameter of the blood vessel [25] and as a result, due to the differences in the diameters of the uterine and testicular artery rings used in this study (up to 1570μm and 716μm, respectively), a direct comparison with uterine tissue of the proportion of α_2_-adrenoceptors was not appropriate.

The contractile response produced by both uterine and testicular arteries to 5-HT, the naturally occurring ligand which acts at both 5-HT_1_ and 5-HT_2_ receptors [13], demonstrates the presence of these receptors in both tissues. Many studies have shown the presence of 5-HT receptors in various arterial beds [26]. As no significant difference was observed between EC_50_ values for both uterine and testicular arteries, it appears that the potency of 5-HT is similar in both tissues. The variation in maximum response between the two tissues, however suggests that 5-HT administration is less efficacious in causing vascular contraction in uterine tissue. It has previously been established that blood 5-HT levels are positively influenced by oestradiol presence and it has been demonstrated that ovariectomy reduces blood 5-HT levels [27]. These results may suggest that canine uterine tissue might exhibit desensitisation of 5-HT receptors as a result of the presence of oestrogen pre-neutering causing a prolonged exposure to 5-HT [13]. One human study evaluating uterine artery contractility to sumatriptan, a 5-HT_1_ receptor agonist [28], showed that tachyphylaxis occurred in uterine arteries and also showed that this does not occur with cerebral arteries [28].

An alternative explanation for the apparent difference in response to 5-HT may be associated with temperature effects. It should be noted that testicular arteries are accustomed to being in a lower temperature environment than uterine arteries. Blood vessels at the extremities, such as cutaneous arteries and veins, that play a role in thermoregulation often exhibit temperature-dependent increases vasoconstriction mediated by adrenergic and/or serotonergic receptors. For example, small arteries within the temperature-sensitive tissues of the equine hoof exhibit cooling-enhanced contraction to 5-HT [29]. The current experiments were all conducted at 37°C, although the in vivo outer temperature of testicular arteries may be slightly cooler. The effects of temperature on testicular artery contractility deserve further investigation.

Another potential explanation for this variation in maximum response is the affinity of 5-HT for α-adrenoceptors [30]. However, although the non-selective α-agonist was more potent in testicular tissue compared to uterine tissue, the maximum responses were similar. Observing the concentration-response curve to 5-HT in the testicular arteries, it could potentially be fitted to a biphasic model, with an EC_50_ of the first phase of 7.1 and the second phase of 5.3. This would give a maximum response for the first phase closer to that of the 5-HT_2_ receptor selective agonist, αmethyl 5-HT. However, both biphasic and monophasic curves had a similar goodness of fit (r^2^ 0.74 for fitting to the biphasic model compared with 0.73 for the monophasic model) and with the sample size in the current study it was not possible to determine whether there may be a distinct second phase. An alpha antagonist, such as benextramine, would need to be used in order to investigate this further [30].

The 5-HT receptor population present in the uterine and testicular arteries was further characterised, focussing mainly on the testicular arteries given the more marked contractile response to 5-HT. The administration of α-methyl 5-HT, a 5-HT_2_ agonist [31], produced a similar dose-response curve to that of 5-HT in the testicular arteries, with a slightly lower maximum response. In contrast, the administration of 5-CT, a 5-HT_1_ agonist [32], failed to produce a vasoconstrictive response. Similarly, it failed to produce a response in the uterine arteries. These results, therefore, suggest that 5-HT_2_ receptors are the predominant receptor subtype causing vasoconstriction in these blood vessels. It has been shown by numerous studies that vasoconstriction induced by serotonin is predominantly mediated via 5-HT_2_ receptors, while vasodilation is sometimes mediated via 5-HT_1_ receptors [33–35]. Human uterine artery studies have also concluded that vasoconstriction is mainly mediated by 5-HT_2_ receptors [34]. There are some exceptions however, such as equine digital arteries and veins where both 5-HT_1_ and 5-HT_2_ receptors cause vasoconstriction [36].

For the teaching of students in the biological sciences, pharmacology, veterinary or medical education programs, having the students observe first-hand the contraction of blood vessels and the effect of G protein-coupled receptor activation and blockade is a very valuable learning experience. These practical activities greatly enhance their understanding of the effect of different types of antagonists and other physiological modulators on the dose-response relationship to agonists. Given the great importance now placed on ‘the 3Rs’ in modern university teaching programs worldwide, the use of these discarded canine tissues may replace the need to kill rodents, rabbits or guinea pigs to obtain the tissues necessary for these teaching practical classes.

In conclusion, this study has characterised the adrenergic and serotonergic receptors mediating vasoconstriction in canine uterine and testicular arteries. These results validate the use of discarded tissue from routine canine neutering procedures as a legitimate and effective tissue source for teaching, and there is promising potential for future application more widely.

## Supporting information

S1 File Spreadsheet containing raw data from blood vessel experiments. (XLSX)
